# Supplementary material for: Are carbon emissions trading and green financial instruments synergistic? -Comprehensive quantitative research based on content analysis
Source: PLoS One. 2024 Mar 7;19(3):e0298601. doi: 10.1371/journal.pone.0298601 (PMC10919601; doi:10.1371/journal.pone.0298601)
Supplement: S1 Data — (ZIP) [file pone.0298601.s001.zip › Data/地级市Z_F工作报告.zip!/─■╧─/╬Γ╓╥╩╨/└┤╫╘╣1⁄2╓┌║┼í╛┬φ┐╦╩2╛▌═°í┐_2005.docx]

各位代表：

现在，我代表市人民政府，向大会作政府工作报告，请予审议，并请市政协各位委员和其他列席人员提出意见。

一、2004年政府工作的回顾

2004年是本届政府任期的第一年，也是我市发展史上较为重要的一年。面对经济社会发展格局因行政区划第二次大调整而发生重大变化的考验，各级政府和全市人民在自治区党委、政府和市委的正确领导下，以邓小平理论和“三个代表”重要思想为指导，全面贯彻落实党的十六大和十六届三中、四中全会精神，牢固树立和落实科学发展观、正确政绩观，解放思想，锐意进取，开拓创新，加快发展，改革开放和现代化建设取得了新的成就，圆满完成了市二届人大一次会议确定的各项目标任务，实现了本届政府工作的良好开局。主要表现在：

(一)经济实力持续提升。2004年，全市完成地区生产总值107亿元，比上年增长13%，第三次突破百亿元大关；地方财政收入5.1亿元，可比增长12.3%。城镇居民人均可支配收入6400元，增长8%，农民人均纯收入2480元，增长9%。工业经济增势强劲。全市工业完成产值170亿元、增加值52亿元，分别增长18%和17.3%，对经济增长的贡献率达到61%。其中，地方工业完成产值61.4亿元、增加值18.5亿元、利润2.7亿元，分别增长22.2%、21%和21.7%，位居全区前列。全市确定的73个投资500万元以上的新建、技改、扩建项目，有64个开工建设，40个建成投产，完成投资19.6亿元，新增产值18亿元。夏进公司、宇华纸业等14家地方优势骨干企业完成产值20.8亿元，实现利润1.44亿元，分别占地方工业的33.9%和51.4%。农村经济稳步发展。粮食生产呈恢复性增长。全年粮食总产66.4万吨，增长3.5%；完成农业总产值27亿元，增长8.5%。农业特色优势产业规模不断扩大。全市奶牛存栏8.6万头，增长47.6%；羊饲养量337万只，增长11.3%；设施瓜菜4万亩，增长25.2%；马铃薯40万亩，增加10万亩；酿酒葡萄4.5万亩，新增1万亩；中药材3万亩，新增1.2万亩。产业化进程加快。夏进乳业、万胜生物、御马酒业等龙头企业实力壮大，新上了米来生物公司谷氨酸、宇华肉食品有限公司和荣宝园肉联厂牛羊肉加工等项目。农业生产条件不断改善。实施了惠农渠与汉延渠上段合并工程、黄河标准堤防建设、苦水河治理、山区人畜饮水、淤地坝等农业基础设施建设重点工程。第三产业持续兴旺。全市实现社会消费品零售总额21亿元，增长10%；居民消费价格总水平104%，上涨4个百分点。城乡居民储蓄存款余额115.8亿元，增长8.3%。建材、家电、农机、农用物资交易及电信通讯、商贸流通、餐饮、旅游业保持了较好发展势头。全市各旅游景点接待游客35万人次，实现收入6300万元，可比增长18%。房地产业增势强劲。全市新开工及接转上年住宅建设170万平方米，已建成110万平方米，完成投资12亿元。房地产交易量7亿元，增长35%。

(二)城乡建设稳步推进。全市共安排城市建设项目213个，完成投资24.8亿元。其中市区重点建设项目56个，完成投资13.2亿元。各县(市、区)对城市主要街区进行大规模的改造，开工建设了157个重点项目，完成投资11.6亿元。青铜峡城市道路与公园建设，同心住宅小区及商贸市场建设，盐池中小学改扩建及住宅开发，红寺堡城区供排水及罗山商城建设等，集中体现了各地城市建设的魄力和亮点。市区按照建设滨河生态城市的总体目标，一手抓规划，一手抓建设。吴忠市城市总体规划(2003—2020年)方案已经自治区人民政府批准，“一城三区”的城市框架正式确定。滨河开发与旧城区改造同步推进，开工建设城市道路10条，总长7.2公里。文卫路北段、吴灵路西段、明珠西路、古城路、新村南路等已完工，完成投资6400万元。实施了供水、供热管网改造、秦渠南集中供热、热计量户表改造等工程，完成投资5360万元。开工建设了劳动力市场和信息网络中心等25个公建项目，市行政中心、财政公积金大楼等20项工程建成使用。新建续建了东方花园二期、世纪嘉园、古城庄点改造等12个住宅开发项目，完成投资9.6亿元，建成面积98万平方米。完成了滨河大道一期4公里标准堤防工程、路面硬化工程及“吴忠情韵”等14个景点建设和1795亩绿化任务，古城湾怡养园等城市绿化、美化项目进展顺利，新增城市公共绿地38.2万平方米，人均增加2.4平方米；城市化率达到32.4%，提高了2个百分点，滨河生态城市的框架初步显现。完成了辖区内同心至沿川子高速公路过境段、国道307线等322公里国道、省道改扩建工程，完成投资5.7亿元。开工建设了大泉至新庄集等26条348.8公里的县、乡、村道路，完成投资1.57亿元，进一步改善了交通基础设施。

(三)“平台”建设初见成效。根据新的市情，充分利用太阳山、牛首山、贺兰山东麓“三山”荒地及资源优势，统筹山川发展，实施园区上山、工业上山，打造发展平台。集中精力实施了太阳山移民开发区“一号工程”，开发区的规划编制和申报基本完成，绿化用地的渠系配套及镇区道路、矿山公路、人畜饮水工程已建成使用，镇政府办公楼附属用房等7个项目全部开工，完成投资1470万元。吸引了区内外许多大型企业前来洽谈投资，第一宗煤炭资源探矿权的招拍挂正依法进行。青铜峡新材料产业基地有32家企业申请进驻，7家开工建设，完成投资1.2亿元。牛首山镁合金产业园区引进8家企业，完成投资8500万元。各县(市、区)工业园区也呈现出良好发展势头。全市7个园区完成基础设施投资7320万元，入驻企业111家，开工项目63个，完成投资6亿元，实现产值1.9亿元。

(四)招商引资取得突破。全面推进对外开放和招商引资，深入开展“项目建设年”和“招商引资年”活动，出台了《关于进一步加强招商引资工作的意见》及其配套文件，实行了目标责任管理，激发了全市上下抓项目、促招商的活力。组团参加了第八届中国东西部合作暨投资贸易洽谈会、宁夏洽谈会等大型节会，赴东部沿海发达地区开展招商引资，邀请佛山市党政代表团来吴开展经贸活动，接待了大量的投资商前来考察。全市签定招商引资项目203个，协议总投资47.6亿元，实际到位资金16.6亿元，分别是上年的4.9倍和5.8倍。荣盛公司冶炼、宁鲁石化一期等64个项目建成投产；宁夏金顺集团10万吨硅铁、盐池富源公司煤炭开发等117个项目正在建设。

(五)社会事业协调发展。实施积极的就业再就业政策，多渠道、多形式安置下岗失业人员3232人，“4050”人员再就业1437人。城镇登记失业率控制在4.3%以内。实施以农民科技培训和农村劳动力转移培训为重点的“百万农民培训工程”，培训农民20万人次。加大劳务输出力度，输出劳务14.4万人次，创收4.5亿元。社会保障体系进一步完善，“两个确保”和“三条保障线”得到落实，全市基本养老保险、医疗保险、失业保险新增参保人数分别达到2900人、6000人和6095人，工伤保险4000余人。切实维护农民工合法权益，清欠农民工工资2480万元，为4100名农民工追讨工资431.8万元。积极解决失地农民生产生活及补偿安置问题，兑付拆迁补偿安置资金4550万元。加强救灾能力体系建设，千方百计保障城乡困难群众特别是灾区群众的基本生活，发放救灾款、粮794万元，社会救助金453.8万元。扶贫开发有了实质性进展，争取扶贫资金2743万元，集中帮扶了75个重点贫困村，贫困人口下降11.5个百分点。扎实开展城市低保扩面提标工作，实现了应保尽保。制定实施《吴忠市创建双拥模范城三年规划》，军政、军民关系进一步密切。

深入实施“人才兴市”战略，继续推行科技特派员创业行动，加快以生产力促进中心、技术创新中心、信息中心为主的科技服务体系建设，提升了地方科技创新与成果转化能力。宁夏吴忠国家农业科技园区建设取得较大突破，辐射带动作用增强，全市新建各类农业科技园区148个。积极推进教育改革，巩固川区“两基”成果，全力打好山区“普九”攻坚战，盐池县“两基”工作率先在宁南山区8县通过自治区评估验收。创建了23所市级示范中小学，新建了盐池五中、同心四中、红寺堡二中等一批危改、百标工程，吴忠中学新校区已投入使用。启动建设了青铜峡体育场、利通区游泳馆、市宣传文化体育中心等基础设施。推广了利通区“宣传文化体育中心户”建设经验，创建了518户农村宣传文化体育中心户。

认真贯彻落实《公民道德建设实施纲要》，积极开展了“全国双拥模范城”和“文明城镇”创建活动，组织开展市直机关首届运动会、“吴忠好地方”巡回展演等群众性文化体育活动，丰富和活跃了城乡文化生活。挖掘吴忠历史文化积淀和弘扬现代文化，成功举办了吴忠历史文脉学术研讨会、宁夏吴忠(青铜峡)高峡长河旅游节、中国西部乳都文化节和青铜峡御马葡萄酒文化节。树立了“人民优秀调解员”康有喜先进典型，深入开展向牛玉儒、任长霞等先进模范人物学习活动，广大干部群众的精神面貌有了新的变化。加强了公共卫生应急体系建设，提高了应对重大突发公共卫生事件的能力。建成了市疾控中心、市医院传染病区、紧急救援中心和一批县级卫生防疫站，城乡群众就医条件得到改善。地震、广播电视、人防、残联等社会事业得到进一步发展。

重视和推进经济与人口、资源、环境协调发展。大力实施“少生快富”工程，控制人口增长，人口自然增长率控制在13‰以内。贯彻落实国家环保法律法规和政策，认真开展清理整顿不法排污企业，保障群众健康环保严查行动等专项治理，环境质量逐步改善。依法保护与合理利用土地、矿产、水资源，深入开展土地市场治理整顿，加大对耕地的保护力度，实现了耕地总量的占补平衡。

(六)社会秩序稳定和谐。全面落实社会治安综合治理各项措施，深入开展“四五”普法，承办了全区基层派出所和司法所建设现场会，推进了基层公安和司法工作。筹集资金120万元，设立了见义勇为奖励基金。重视群众的来信来访，落实责任，把矛盾化解在基层和萌芽状态。深入开展“严打”整治斗争，有力地打击了各种违法犯罪活动。积极构建全方位、多层次的社会治安防控网络体系，组建了治安联防队伍，整合基层派出所警力资源，增强了社会面的控制能力，人民群众的安全感进一步增强。认真贯彻落实党的民族宗教政策，召开了全市首届民族团结进步表彰大会，依法管理民族宗教事务，保持了民族宗教领域的稳定。扎实开展市场经济秩序整顿和规范工作，严厉打击各种扰乱市场经济秩序的违法活动，市场经济秩序进一步净化。强化安全生产监督管理，依法加大对道路交通、非煤矿山等重点行业和领域的监督检查力度，遏制重特大事故发生，全市安全生产形势基本稳定。

(七)政府工作全面提速。加强政治文明建设，以建设责任政府、有限政府、法治政府、亲民政府为目标，深入开展公务员素质提高年活动，加快推进政府职能转变。加强制度建设，促进政府工作的程序化、规范化。一年来，市政府及其工作部门制定细化工作制度270个，特别是制定出台了《吴忠市人民政府工作规则》等相关制度，依法规范了行政程序。全面深化行政审批改革，清理行政许可实施主体35个，保留行政许可事项203项。深入推行政务公开，进一步完善了政务大厅、服务窗口和政务公开栏工作制度和程序，建立完善了公开办事制、首问责任制、限时办结制、服务承诺制、岗位责任制、督查反馈制等制度，对涉及行政审批、登记、备案、核准等事项限时办理。认真贯彻《全面推进依法行政实施纲要》，成立了政府法律顾问团，明确了政府各部门在依法行政进程中的主要任务和措施，严格按照法定权限和程序行使权力、履行职责。自觉接受人民代表大会及其常委会的依法监督和人民政协的民主监督，认真办理人大代表议案、建议和政协委员提案。建立了政府与工会的联系制度，广泛听取各方面的意见。依法行政的意识增强，行政权力的运行趋向规范，监督约束机制初步形成，办事效率明显提高。

各位代表！实践充分证明，只要始终坚持以邓小平理论和“三个代表”重要思想为指导，善于解放思想，更新观念，抢抓机遇；始终坚持树立和落实科学发展观，善于立足市情，科学决策，统筹兼顾；始终坚持立党为公、执政为民，善于以人为本，凝聚民心，形成合力；始终坚持发展第一要务，善于开拓创新，锐意进取，干事创业，我们就一定会排除万难，无往不胜，我们的事业也一定会蒸蒸日上、兴旺发达、硕果盈枝！总结过去一年的工作，我们也深深体会到，一切成绩的取得是自治区党委、政府的亲切关怀和市委正确领导的结果，是市人大、市政协及社会各界有效监督和大力支持的结果，是全市人民团结拼搏、无私奉献的结果。在此，我代表市人民政府，向为吴忠改革开放和现代化建设做出积极贡献的全市广大工人、农民、知识分子和干部、驻吴部队、武警官兵、公安干警、中央及区属驻吴单位，表示崇高的敬意！向人大代表、政协委员，各民主党派、工商联、人民团体、离退休老同志和关心、支持吴忠发展的社会各界人士，表示衷心的感谢！

回顾过去一年，我们也清醒地看到，我市经济社会发展中依然存在着一些困难和问题，比较突出的是：影响经济发展的结构性矛盾和市场对资源有效配置的体制性障碍依然存在，整体竞争力不强；工业高增速低效益、产业链条短及企业筹资难的问题仍然比较突出；农业受自然灾害影响较大，疫病防治的形势严峻，农业生产成本上升，农业直接收入相对减少，山区脱贫任务仍很艰巨；固定资产投资增幅回落，城市建设融资难度增加，一些基本建设项目开工不足，拆迁安置补偿资金缺口大，失地农民生产生活困难；招商引资的规模较小、层次偏低，经济外向度还不高；财政基础薄弱，历史负担重，收入增长缓慢、刚性支出快速增加，硬性缺口大，对经济的支付调控能力比较弱；个别部门职能转变滞后，服务意识不强，投资环境、发展环境亟待进一步改善，等等。对于上述问题，我们将采取更加积极有效的措施，努力加以解决。

二、2005年国民经济和社会发展的主要目标和任务

2005年是实现“十五”计划目标、衔接“十一五”规划的重要一年。按照市委的总体部署，政府工作的总体要求是：坚持以邓小平理论和“三个代表”重要思想为指导，深入学习贯彻党的十六大和十六届三中、四中全会精神，以加强党的执政能力建设为根本，以“再造新优势、建设新吴忠”为目标，以科学发展观统揽经济社会发展全局，加快推进新型工业化、城市化、农业产业化进程，努力实现全市经济社会全面协调可持续发展。主要预期目标是：地区生产总值达到125亿元以上，增长12%，力争达到13%；全社会固定资产投资75亿元，增长15%；地方财政收入达到4.3亿元，可比增长10%；社会消费品零售总额25亿元，增长11%；人口自然增长率控制在13‰以内；城镇登记失业率控制在4%以内，新增城镇就业10000人；城镇居民人均可支配收入达到6900元，增长6%；农民人均纯收入2600元，增长4‰。全面完成“十五”计划。为此，要突出抓好以下七个方面的工作：

(一)深入实施工业强市战略，强化工业主导型经济增长格局

工业是建设经济强市的主要支撑，也是深化市域经济结构调整、提高产业集中度、培育新的增长点的主要突破口。坚持走新型工业化道路，着力在支柱产业培育、园区建设、项目建设上增补措施，加大力度，务求取得实效。做强优势产业。加快产业结构的跨地区、跨行业调整，集中生产要素和资源配置，提升能源电力、新材料、乳品、葡萄酒、羊绒、建材等对全市经济有重大支撑作用的产业。加快开发与电解铝、金属镁、铁合金、电石、煤炭等产品相关联、相配套的产业，合理利用资源，提高产业集中度，发展循环经济。充分利用现有生产能力及能源、矿产资源丰富的优势，把铝镁产业做为支撑工业发展的拳头产业来抓，在大力支持青铜峡铝业、全世达镁业等铝镁企业发展的同时，依托园区建设，积极新上铝合金、金属镁及镁合金等铝镁精深加工项目，扩大铝镁产业规模。做大园区平台。从战略上调整优化工业布局，引导工业向园区集中，创新园区管理体制和运行机制，集中布局一批重点工业项目。继续把太阳山移民开发区建设作为经济工作的重点，加快矿产资源勘探、采矿权的招拍挂步伐，加大对园区规划及基础设施建设的投入力度。尽快设立太阳山移民开发区政务大厅，实行一站式服务，打造全市招商引资的窗口，以资源优势大招商、招大商，力促区内外大型企业投资置业。加快青铜峡新材料产业基地、牛首山镁合金园区，同心羊绒工业园、金积工业园等园区建设步伐，进一步完善园区功能，提高承载大项目的能力和园区聚集效能，力争有更多的大项目、新项目入园建设，早日投产达标，使园区成为工业发展的最大亮点。突出项目建设。力促天辰公司、宁夏荣盛、同盛冶炼公司等已建成项目达产达效，重点抓好金昱元公司年产20万吨PVC扩建、万胜生物公司30万吨玉米深加工等投资在5000万元以上、对今后全市产业调整具有重大带动力的项目，以及宁鲁石化二期、三期2万吨石化产品扩建、万兴公司3000台扬天挂车及汽车改装等61个投资在500万元以上的技改、续扩建项目建设，完成投资20亿元。通过产业培育和项目建设，带动整个工业升级转型。力争全年完成地方工业产值75亿元、增加值22.5亿元，实现利润3.24亿元，分别增长25%、25%和22%，确保工业经济总量两年增加50%。

(二)切实加强农业和农村工作，努力增加农民收入

深入贯彻中央1号文件精神和中央农村工作会议精神，落实粮食直补、化肥、种子补贴和减免农业税等各项扶持政策，抢抓农业、农村发展的新机遇，更加积极地支持“三农”发展，促进农民收入的有效增长。用抓工业的理念发展农业，用现代农业经营方式和科技成果改造提升传统农业，用持续有效的农业基础设施建设支撑保障农业，提高农业的产出和效益。提升发展基地。坚持以市场为导向，以科技为支撑，以园区为载体，以结构调整为主线，稳定粮食生产，大力发展特色农业、生态农业、绿色农业、效益农业，抓好农业产业化基地建设。集中引导发展乳业、玉米淀粉、羊绒、设施农业、酿酒葡萄、马铃薯、优质米等产业基地，适度扩大生产规模，提高附加值，拉长产业链，最大程度地增加农民收入。力争奶牛饲养量达到10万头、羊饲养量350万只、新增设施农业5000亩、高效经济作物10万亩、优质马铃薯20万亩、酿酒葡萄5000亩。全面实施优质农产品无公害和标准化生产，推进科技特派员创业行动，加快利通区现代农业示范县和宁夏吴忠国家农业科技园区建设，带动农业上水平、增效益。培育壮大龙头。重点支持宁夏新华百货夏进乳品公司10万吨液态奶生产线、宇华公司牛羊肉加工、御马酒业10万吨葡萄酒、米来生物公司20万吨玉米淀粉、绿海公司苜蓿加工、天启薯业马铃薯深加工、涝河桥牛羊肉市场等一批农业产业化龙头项目，提高辐射带动能力。完善增收机制。积极推行农村水费改革，发展节水农业。全面推行农村小额贷款、信用工程建设和农村合作化经营，建立健全动物疫病防疫体系，积极推行农业生产保险，创新农业经营体制，切实解决农民一家一户办不了和办不好的事情。加强农民负担监督管理和农用物资市场监督检查，加强粮食市场监管，保护和调动好农民生产积极性。夯实农业基础。坚持基本农田保护制度，切实保护耕地。认真实施唐徕渠和西干渠合并工程、西夏渠开挖工程，完成黄河标准堤防4.6公里，启动建设黄河护岸林场。实施同心东部30万亩马铃薯种植基地水源工程，加快山区生态环境治理和人畜饮水工程建设，治理山区水土流失。巩固和扩大封山禁牧、退耕还林还草成果，加强农田水利基本建设，实行山、水、田、林、路、村庄综合治理，增强农业发展后劲。加大扶贫力度。实施“整村推进”扶贫规划，着力改善山区生产、生活条件。加强山区劳动力技能培训和劳务输出，突出发展草畜产业，因地制宜发展马铃薯、中药材等退耕还林还草后续产业，切实增加贫困地区群众收入。继续落实扶贫部门、项目、资金和人员，落实帮扶措施，促进山区脱贫致富，力争农村贫困人口大幅下降。

(三)积极推进城市化进程，高标准建设滨河生态城市

加快推进城镇化是实现以城带乡、城乡协调发展的重要动力。坚持把加快城乡建设、打造城市品牌作为促进经济社会与人全面发展的重要内容，综合运用市场机制和经济手段，不断加快城市化进程。科学合理规划城市。牢固树立规划管理的龙头地位，依据新批准的城市总体规划，统筹各区域基础设施规划，尽快组织编制市区近期发展详规、各项专业详规和分区规划，筹建吴忠发展规划展示厅。严格执行市域城镇体系规划，总体兼顾，协调发展，努力建设具有“塞上江南”风貌的新型滨河城市。项目建设带动城市。坚持按照吴忠市区西移和青铜峡市区东扩的城市发展战略，以黄河吴忠市区过境段综合治理为突破口，推进吴青一体化进程。加快滨河大道、吴青路及陈袁滩镇沿线的开发建设，充分保护和建设沿黄河生态自然景观带，注重以生态连接的方式和项目建设拉大滨河生态型城市框架。新建及改造胜利西街、明珠路西段延伸、吴灵路东段改造、西二环向北端直等10条城市道路。新建盛世花园、塞上江南小区、长城花园、颐嘉花园和续建古城庄点改造等13个住宅小区。开工建设秦汉渠办公大楼、烟草专卖局大楼、地税大楼、新生北街商城、新区医院等23个重点项目。启动滨河大道二期生态绿化带、明珠公园、明珠广场改造等12个城市基础设施建设项目。规范管理提升城市。建立健全市、区、乡镇(街道)、社区四级管理体制，加快转变城市管理的机制和方式，注重运用市场手段加快城市绿化、美化、亮化和净化，强化“讲秩序、讲卫生”意识。加快城市垃圾中转站、主要街道公厕建设，加强社区物业管理，开展“物业管理年”活动，不断提升人居环境。着力推进城市投融资体制改革和城市公用事业管理体制改革；积极推进市区收购储备土地的招拍挂，加快土地运作经营步伐，加强城市国有资产管理，发挥国有资产投资经营公司投融资作用，积极探索新的投融资机制，为激活市区建设融集资金，注入动力。城镇扩张聚集城市。充分运用市场机制，促进多元投资，推进县(市、区)城镇建设。加快小坝汽车站与银基花园、盐池安定西街与花马池西街延伸、同心银平街与西北环路、红寺堡市政道路与人民医院等项目建设步伐。继续改造提升主要街区，拉大城市框架，完善基础设施，增强服务功能，提升城市聚集效能。同时，大力发展太阳山、陈袁滩、金积、韦州、花马池、青铜峡镇等中心集镇，完善城镇功能，形成中心城市与中心集镇协调推进、共同发展的格局。交通网络连接城市。加快吴忠市区至青铜峡市区黄河大桥、宁东化工基地(古窑子)经吴忠市区至青铜峡新材料产业基地东西通道的勘测和工程立项，促使中太(银太)铁路、中盐高速公路以及中盐高速公路至太阳山连接线工程早日开工建设，着力实施沿山公路201道改线、吴忠滨河大道二期、三期工程及滨河大道连接线公路建设，构建城市交通框架。同时，加快农村道路基础设施建设，提高公路等级和通达能力，加速城乡一体化进程。

(四)大力发展开放型经济，着力提升招商引资质量

坚持把招商引资作为提升对外开放水平，吸引和扩大投资的重点，引导全市各级组织、干部群众及社会各方面力量面向沿海发达地区、产业特色突出的中心城市，面向国内外知名大企业和大集团开展招商，努力实现引资额度、引进项目、利用外资和出口创汇的新突破。强化招商责任。继续实施招商引资目标责任制，创新招商引资工作思路，完善工作机制和措施，围绕服务客商和引进项目抓落实，努力在全市营造一种人人想招商、时时议招商、处处抓招商的氛围。优化招商环境。牢固树立环境就是生产力、创新环境就是发展生产力的思想，在继续改善硬环境的同时，着力强化软环境建设。注重提高全体市民的内在素质和信用意识，建设“诚信吴忠”，真正在全市形成“人人都是投资环境，事事都关吴忠形象”的思维理念。认真落实各项优惠政策，降低外商投资成本，提高外商投资回报。简化办事程序，提高办事效率，加强在谈项目、签约项目和已进驻客商的跟踪服务，以便捷高效优质的服务吸引客商、感动客商、留住客商，努力实现我市投资环境一年一个样，三年大变样的奋斗目标。创新招商方式。加强招商引资信息库和项目库建设，做好项目的包装对接。充分利用现代招商手段，开辟招商引资的多种有效载体，依托我市农副产品、矿产资源等优势，采取委托招商、中介招商等代理方式，搞好项目招商、以商招商、网上招商、“小分队”招商和定向、定位、定人、定点招商。继续实施“走出去，请进来”战略，对重点企业、重大项目、重要客商连年跟踪，把客商邀请、项目对接、签约合作等工作落实到每一次招商活动中，延伸招商引资触角，拓宽招商引资渠道，提高招商引资质量。扩大招商成果。着眼于提高招商引资项目签约率、资金到位率和投产开工率，着力抓好在谈项目与签约项目的跟踪推进工作。积极落实青铜峡市与上海勤昌实业公司投资2.1亿元汽车轮毂项目、与山东中信贸易公司投资1.3亿元的20万吨玉米淀粉加工项目，利通区与山东天辰有限公司投资1.2亿元的冶炼项目，盐池县原野蜂产品公司与深圳益康元蜂产品公司投资4435万元的蜂胶加工项目，同心石膏粉厂与四川广元金辉事业有限公司投资1亿元的水泥熟料生产基地项目，红寺堡开发区与浙江正日集团投资1亿元的桑蚕基地建设项目。力争年内在能源化工、新材料、农副产品加工等优势产业的招商引资上有新突破，实际到位资金20亿元以上。同时，继续争取国债项目和专项资金，加快城市排污、垃圾处理、乡村公路等基础设施建设；鼓励出口企业扩大生产规模，增加出口量。

(五)全力加快非公有制经济发展，培育新的经济增长点

坚持把加快发展非公有制经济作为强市、强县的重中之重，落实加快发展非公有制经济的政策措施，深入开展“非公有制经济发展年”活动。按照“扩大总量、提高质量”的方针和扶优扶强的原则，着力催生一批，培育一批，引进一批，壮大一批非公有制经济群体。切实打破对非公有制企业的投资限制，鼓励非公有制经济以独资、参股、控股、合作、联营和特许经营等方式，积极参与城乡基础设施和公用事业建设，向教育、文化、卫生、医疗、体育等领域拓展。开展争创非公有制企业十强活动，重点选择100户有一定规模和实力的非公有制企业，从资金、技术、人才等方面给予倾斜，推动非公有制经济由家族式粗放经营向开放型集约经营转变，提高非公有制经济的规模和竞争力。非公有制企业申报的符合国家产业政策的项目，同等享受技改资金、科技三项费用资金、农业产业化资金及财政贴息、财政资助和低息贷款等优惠政策。各级财政每年安排一定的专项资金，用于支持非公有制企业创业发展，力争非公有制经济增幅达到40%以上。加快吴忠市区和银川市第三产业的互动互补、互利互惠，进一步密切联系，借船出海，培育优势。充分发挥吴忠人“有胆识、能吃苦、善经营”的传统，大力发展商贸流通等第三产业，积极推进连锁经营、物流配送和电子商务，以现代营销方式改造提升传统商贸服务业，利用现有优势，出台优惠政策，整合各类商贸市场，做大做强做活市场，建设高标准、大规模、西部一流的农牧产品批发市场，重振吴忠“旱码头”雄风。学习借鉴义乌人的创业精神、经商理念和经营管理市场的经验，加强合作与交流，努力把吴忠打造成宁夏乃至“西部的义乌”。继续巩固发展旅游、餐饮、房地产等产业，大力发展邮政、电信、信息咨询、金融、保险、社区便民服务等现代服务业，力争使非公有制经济成为推进第三产业发展的主力，使第三产业成为市域经济发展的重要动力。

(六)全面发展社会事业，努力构建和谐社会

坚持以人为本，促进经济与社会协调发展。深入落实促进就业再就业的政策措施，积极推进下岗失业人员再就业。大力发展劳务经济，力争劳务输出稳定在15万人（次）以上。进一步完善社会保障体系建设，积极扩大基本养老保险、基本医疗保险、失业保险、工伤保险的覆盖面，启动生育保险。完善城乡社会救助体系，切实保障困难群众生产生活。积极筹措资金，妥善安置失地农民。大力加强财源建设，确保财政收入稳定增长。按照建立公共财政框架的要求，深化财政管理改革，完善部门预算制度、综合财政预算制度、专项资金专款使用制度、政府采购制度、国库集中收付制度；加强财政支出管理，提高财政资金使用效益。

深入实施科教兴市和人才强市战略，加强科技创新体系建设，努力提高科技支撑的水平和科特派创业水平，示范推广10项科技重大项目、100项科技发展项目、60项先进适用技术。坚持教育优先发展战略，完善“以县为主”的农村义务教育管理体制，巩固提高川区“两基”水平，切实推进山区“普九”攻坚步伐，确保红寺堡开发区如期通过自治区评估验收。优化教育结构，实行办学模式多元化，推进联合办学，支持社会力量办学。推进素质教育，加快普及高中阶段教育，重视并加强民族教育、幼儿教育和特殊教育，积极发展职业教育、远程教育，提高教育质量。建成吴忠职业技术学院综合楼、实训楼，青铜峡高级中学、利通区第三中学迁建等25个国债、百标、危改等建设项目。

坚持先进文化的前进方向，进一步加强公民道德建设，继续广泛开展创建“文明城镇”，“双拥模范城”等活动，全力做好全市农村宣传文化体育中心户建设工作，深入开展群众性文化活动，大力发展文化产业。深入挖掘吴忠历史文化，努力繁荣吴忠民族文化、大力弘扬吴忠现代文化，提升城市文化品位，扩大吴忠的知名度和影响力、着力塑造特色浓郁的“黄河金岸、水韵吴忠”新形象。加强文化市场管理，完善文化网络，重视农村文化和社区文化建设。积极筹建吴忠历史博物馆和青少年革命传统教育基地，建成吴忠体育馆、书画院。举办吴忠市首届运动会，广泛开展全民健身运动。继续加快公共卫生体系建设，建立健全市、县(市、区)、镇(办、社区)、村四级疾病防控网络，完善疾病控制、医疗急救、卫生监督体系。加强农村医疗卫生工作，认真做好青铜峡市农村新型合作医疗制度试点工作，探索提高农村疾病防控能力和人民健康水平的有效途径。

正确处理经济发展与人口、资源、环境的关系。加强人口与计划生育工作，搞好优质服务和综合改革，建立完善计划生育利益导向机制和保障机制，提高人口素质，优化人口结构。稳定川区低生育水平，依法加强山区人口管理，严格控制人口增长。强化安全生产监督管理，落实安全生产行政责任追究制，确保全市安全生产形势平稳。依法保护、合理开发和节约使用土地、水、矿产等各种不可再生资源，严厉打击私开乱挖等非法采矿行为，提高资源的永续利用水平。继续贯彻落实环境保护基本国策，加大环境污染治理和保护力度，建设市区空气自动监测站；认真实施《环境影响评价法》，加强资源开发和建设项目环境保护管理；积极推进循环经济和环保产业发展，增强全民环保意识，确保环境安全。

加强精神文明和民主法制建设，促进“三个文明”全面发展。继续贯彻实施《公民道德建设实施纲要》，加强社会公德、职业道德和家庭美德教育，提高广大市民的综合素质。广泛开展创建文明城市、文明行业、文明村镇、文明社区活动，巩固提高文明创建工作成果。加强国防教育和民兵预备役建设，扎实做好“双拥共建”工作，积极为部队办实事、解难题，巩固新型军政军民关系。认真执行人民代表大会及其常委会的决定决议，自觉接受监督。进一步加强与人民政协的联系，支持人民政协履行好政治协商、民主监督、参政议政的职能。认真办理人大代表议案、建议和政协委员提案。主动听取各民主党派、工商联和社会各界的意见建议。切实加强基层民主政治建设，推进政务、村务、厂务、校务公开，提高执政水平和透明度。认真贯彻落实民族宗教政策，大力发展民族团结进步事业，依法加强对宗教事务的管理，维护民族宗教领域稳定。扎实做好信访工作，强化信访责任制，积极排查化解各类社会矛盾，妥善处理信访突出问题及群体性事件。继续推进社会治安综合治理，深入开展 “平安吴忠” 创建活动，深化“严打”整治专项斗争。严厉打击各种刑事犯罪活动，坚决扫除“黄、毒、赌”等社会丑恶现象，维护社会稳定。继续加强市场经济秩序整治，严厉打击制售假冒伪劣产品的违法行为，维护消费者合法权益。关心支持老龄、残疾人事业，继续做好外事侨务、防震减灾、气象预报等事业。

(七)切实加强自身建设，服务经济社会发展

按照“为民、务实、高效、清廉”的要求，加快职能转变和管理创新，切实提高行政效能和服务质量，更好地履行经济调节、市场监管、社会管理、公共服务的职能。以建设法治政府为总抓手，切实提高行政能力。

坚持与时俱进，解放思想。坚定不移地贯彻执行党的基本路线、方针和政策，不断提高工作水平和解决实际问题的能力。坚持用辩证的观点和发展的眼光看待和解决前进道路上的困难，站在更大范围、更高层次上谋划发展，在纷繁复杂的局面中突破主要矛盾，带动整体工作上水平。牢固树立创新意识，坚决冲破一切影响改革与发展的传统观念、保守意识和守成思想，大力弘扬勇于进取、乐于奉献、敢于超越的时代精神，在实践中不断创新思路、创新体制、创新机制，努力开拓思想解放的新境界。

加快推进政府职能转变。充分发挥市场在资源配置中的基础性作用，将更多的财力、物力用于加强公共设施建设和提供优良的公共产品，把政府职能转到为市场主体服务和创造良好发展环境上来，让广大群众共享发展成果，共享文明生活，努力构建和谐社会。切实加快政府信息化建设，大力开发信息资源，推行电子政务，提高行政效率。建立健全各种预警和应急机制，提高政府应对突发事件和各种风险的能力。

不断增强依法行政能力。深入贯彻实施《行政许可法》，深化行政审批改革，将保留的行政许可项目，全部纳入规范化、制度化管理轨道。大力推行“阳光工程”，规范政务大厅运行机制，行政许可清理后保留的项目以及与企业和群众关系密切的程序性项目、备案项目、公益性服务项目全部进驻政务大厅，努力实现“一门受理，全程代理”，实行集中限时办理，方便群众办事。强化行政执法监督检查，落实执法责任制和过错责任追究制，加强对规范性文件的备案审查，发挥行政复议、行政诉讼监督作用，坚决纠正行政违法行为，依法保护公民、法人或其他组织的合法权益，加快建立权责明确、行为规范、监督有效、保障有力的行政执法体制。

着力提高政府决策水平。坚持和完善政府和领导干部联系群众制度、人民群众建议征集制度、社情民意反映制度、与群众利益密切相关的重大事项社会公示和听证制度。建立和完善公众参与、专家论证和政府决策相结合的行政决策机制，完善和坚持行政决策程序，切实做到依法决策、科学决策和民主决策。对涉及经济社会发展全局、涉及公共利益的重大问题和事项，要深入调查研究，广泛听取意见，充分进行论证，主动接受群众监督，真正体现群众意愿，维护人民群众的根本利益。

进一步提高机关工作效率和服务水平。进一步改进政府工作流程，坚持办文办事限时制，简化办事程序，切实减少会议、文件和不必要的检查、评比活动。建立健全有利于提高工作效率和服务水平的各项工作制度，强化目标管理考核，加大行政督查力度，用制度规范行政行为、推进工作落实的长效机制。政府各部门要加强协作，密切配合，杜绝推诿扯皮、敷衍塞责现象，确保政令畅通。

大力弘扬求真务实作风。牢固树立科学发展观和正确政绩观，坚持讲实话，出实招，办实事，求实效，把工作的着力点放到研究解决改革发展稳定中的重大问题上，放到研究解决群众生产生活中的实际困难和问题上。坚持立党为公、执政为民，大力倡导为民务实的执政风格，切实维护人民群众的根本利益。狠抓党风廉政建设，全面落实党风廉政建设责任制，坚决查处违纪违法案件，坚决纠正部门和行业不正之风，坚决纠正损害群众利益的不正之风。加强行政监察和审计监督，从源头上预防和治理腐败。

今年还有一项重要任务，就是抓紧做好“十一五”规划和2020年远景目标的制定工作。做好这项工作，对于贯彻落实科学发展观，统一各方面认识，团结广大干部群众，加快经济社会发展，具有重要意义。要深入研究我市改革发展稳定出现的新情况、新特点、新问题，努力把握经济社会发展的新趋势，加强调查研究，充分发扬民主，切实提高规划的针对性、科学性、预见性和指导性。要加强统计工作，搞好经济普查，为制定“十一五”规划提供科学依据。

各位代表！

肩负着强市富民的时代重任，我们又踏上了新的征程。形势催人奋进，发展重在践行。让我们紧密团结在以胡锦涛同志为总书记的党中央周围，在自治区党委、政府和市委的坚强领导下，紧紧依靠全市人民，以更足的干劲，更实的作风，努力夺取今年经济建设和社会各项事业的新胜利，共同创造更强的吴忠、更美的城市、更好的生活！
